# CD21^–^ CD27^–^ Atypical B Cells in a Pediatric Cohort Study: An Extensive Single Center Flow Cytometric Analysis

**DOI:** 10.3389/fped.2022.822400

**Published:** 2022-06-03

**Authors:** Francesco Corrente, Sara Terreri, Patrizia Palomba, Claudia Capponi, Mattia Mirabella, Carlo Federico Perno, Rita Carsetti

**Affiliations:** ^1^Microbiology and Diagnostic Immunology Unit, Bambino Gesù Children’s Hospital, IRCCS, Rome, Italy; ^2^Diagnostic Immunology Research Unit, Multimodal Medicine Research Area, Bambino Gesù Children’s Hospital, IRCCS, Rome, Italy; ^3^Multimodal Medicine Research Area, Bambino Gesù Children’s Hospital, IRCCS, Rome, Italy

**Keywords:** flow cytometry, B cell subsets, immunodeficiencies, autoimmune diseases, inflammatory diseases, hematological disorders, atypical B cells, CD21 B cells low

## Abstract

Atypical B cells (atBCs) are a distinct B-cell population and represent approximately 5% of B cells in peripheral blood (PB) of healthy adult individuals. However, in adults these cells are expanded in conditions of chronic infections, inflammation, primary immunodeficiencies, autoimmune diseases, and aging. Their immunophenotype is characterized by the lack of CD21 expression and the hallmark human memory B-cell marker CD27. In this study, we investigated the immunophenotype of atBCs in different pediatric pathological conditions and correlated their expansion with the children’s clinical diagnosis. We were able to retrospectively evaluate 1,571 consecutive PB samples, corresponding to 1,180 pediatric patients, by using a 9-color flow-cytometric panel. The results, compared with a pediatric healthy cohort, confirmed an expansion of atBCs in patient samples with percentages greater than 5% of total B cells. Four subpopulations with different expressions of IgM and IgD were discriminated: IgM^+^IgD^+^, IgM^+^-only, IgD^+^-only, and IgM^–^IgD^–^. IgG^+^ atBCs were predominant in the IgM^–^ IgD^–^ subpopulation. Moreover, the study highlighted some features of atBCs, such as a low CD38 expression, a heterogeneity of CD24, a high expression of CD19 and a large cell size. We also demonstrated that an increase of atBCs in a pediatric cohort is correlated with immunodeficiencies, autoimmune, inflammatory, and hematological disorders, consistent with previous studies mainly performed in adults. Furthermore, our flow cytometric clustering analysis corroborated the recent hypothesis of an alternative B origin for atBCs.

## Introduction

For over a decade, a particular subset of mature B cells, lacking the expression of CD21 (complement receptor type 2) and the hallmark human memory B-cells (MBCs) marker CD27, has been the focus of increasing interest. Several names, based on characterizing markers or discovery contexts, have been used to describe these cells: atypical memory B cells, age associated B cells, tissue-like memory B cells, CD11c^+^ B cells, T-bet^+^ memory B cells, double-negative CD27^–^ IgD^–^ B cells, or CD21^–/low^ B cells (1–6). These cells are normally found in peripheral blood (PB) of healthy adult individuals, where they represent approximately 5% of the total B cells (2). Despite the lack of CD27, the expression of markers indicative of antigen experience, such as CD44, CD69, CD80, CD84, and CD86, and the evidence of an extensive somatic mutation, suggest that these atypical B cells belong to the memory compartment (7). Furthermore, stimulation with a combination of B cell receptor (BCR), Toll-like receptor (TLR) 7/8 and interleukin (IL)-2 induced proliferation and differentiation of the CD21^–/low^ B cells that are comparable to those observed in CD21^+^ CD27^+^ memory B cells (2). A recent study based on single-cell RNA sequencing showed that a population of atypical CD21^–^ CD27^–^ B cells is part of an alternative lineage of B cells that participates in normal responses to vaccination and infections in humans (8).

In the study, we refer to this CD21^–^ CD27^–^ B-cell subset as atypical B cells (atBCs). An increase of atBCs has been attributed to chronic immune activation and inflammation and seems to contribute to deficiencies in the acquisition of immunity by a yet unclear mechanism common to a variety of chronic infectious diseases, such as HIV, *Plasmodium falciparum*, *Mycobacterium tuberculosis*, and Hepatitis C virus infections (9). However, the role of atBCs in the immunity of chronic infectious diseases is still controversial (10). An association between atBCs and splenomegaly and autoimmune cytopenia in patients with common variable immunodeficiency (CVID) has also been described (11). Based on these studies, the presence of over 20% atBCs has been proposed as a criterion for the classification of CVID patients prone to develop autoimmunity (12). In the peripheral blood of patients with severe autoimmune diseases, such as rheumatoid arthritis (13, 14), systemic lupus erythematosus (15) and Sjögren’s syndrome (16), atBCs are expanded. This B population, containing autoreactive or polyreactive clones, is refractory to BCR stimulation and responds robustly to TLR activation (17). It has been suggested that atBCs localize in the sites of inflammation where they may be activated by inflammatory mediators, may expand and potentially contribute to the pathology of the autoimmune disease (17). Furthermore, an abundance of atBCs has been shown to be one of the key age-related changes in the B-cell compartments. Their increasing prevalence with age contributes to several well-established features of immunosenescence, including reduced B-cell genesis and damped immune responses (3). Moreover, hematological patients with elevated atBCs, who underwent allogeneic hematopoietic stem cell transplantation (HSCT), presented a higher risk of developing chronic graft-versus-host disease (cGVHD) (18).

To date, most of the research and published data on atBCs have been performed on adult patients, whereas very little is known about the distribution and the immunophenotype of atBCs in pediatric diseases. In this study, we present an extensive flow cytometric analysis carried out on B-cell subsets in a heterogeneous pediatric cohort, with a specific focus on the atBC population. The main purpose of this research is to investigate the correlation between pediatric diseases and the increase in prevalence of atBCs.

## Materials and Methods

### Study Design

We performed a retrospective immunophenotypic analysis of 1,571 consecutive fresh PB samples, corresponding to 1,180 pediatric patients (261 patients were evaluated more than once) collected at the Bambino Gesù Children Hospital in Rome from June 2019 to December 2020. Immunophenotypic analysis were carried out on all PB samples, whereas the correlations with clinical features were only performed on the first flow-cytometric assessment. Seven categories of disorders were identified based on the main clinical findings, diagnosis, or presumptive diagnosis of the patients: immunodeficiency disorders, autoimmune diseases, inflammatory diseases, hematological diseases, infectious diseases, neurological diseases, and other pathologies. Some patients had been diagnosed before the study. We have also included 50 age-matched healthy children control. In the study cohort, 58.1% of the children were males and 41.9% were females with a median age of 6 years (range: 0–18 years); in the healthy cohort, 62% of children were males and 38% were females, with a median age of 5 years (range: 0–18 years).

### Flow Cytometry Analysis

In order to achieve high levels of standardization, reagents of a B-cell multicolor panel were used in a dried format (BD Bioscience, Table 1) and bulk lysing was performed on the entire blood sample. Briefly, 500 μl of fresh total PB (EDTA) were incubated for 10 min at room temperature with 9.5 ml of the lysing solution Pharm Lyse 1X (BD Biosciences) to lyse red blood cells. Cells were then washed twice with 10 ml of phosphate-buffered saline (PBS) containing 1% of bovine serum albumin (BSA) to wash-off the free IgGs which could interfere with the anti-IgG antibody surface staining, preventing the identification of IgG^+^ memory B cells. Cells were re-suspended in 200 μl of PBS and added to a B-Cell lyotube (BD Biosciences). The CD21 BV605 antibody was also added to the mix. After a 20 min incubation at room temperature in the dark, samples were washed with PBS 1% BSA and re-suspended in 300 μl PBS 1% BSA.

**TABLE 1:** Antibodies used for the staining of the peripheral blood and the identification of B-cell subsets.

Marker	Fluorochrome	Clone	Cod. n°
CD45	V500-C	2D1	
CD19	PE-Cy7	SJ25C1	
CD24	PE	ML5	
CD27	APC	L128	626220 (Lyotube)
CD38	PerCP-Cy5.5	HIT2	
IgM	FITC	G20-127	
IgG	APC-H7	G18-145	
IgD	V450	IA6-2	

CD21	BV605	B-ly4	740395

Flow cytometric data were acquired on a BD FACSLyric™ cytometer (BD Biosciences) and analyzed by FlowJo ver. 10.7 (Becton Dickinson and Company). Acquisition criteria were set as follows: 30,000 CD19^+^ events OR max 3 min acquisition at medium rate (60–70 μL/min). Samples with less than 3,000 CD19^+^ events were excluded from the analysis. CD19^+^ B-cells were gated into several subpopulations: transitional (CD24^+^CD38^++^) and plasmablasts (CD24^–^CD38^++^), naïve [NOT (transitional or plasmablasts) CD21^+^CD27^–^], memory B cells (MBCs) [NOT (transitional or plasmablasts) CD21^+^CD27^+^], activated memory B cells (actMBCs) [NOT (transitional or plasmablasts) CD21^–^CD27^+^], and atypical B cells (atBCs) [NOT (transitional or plasmablasts) CD21^–^CD27^–^].

### T-SNE and FlowSOM Analysis

Selected fcs files from 18 pathological and healthy patients were imported into FlowJo ver. 10.7 and CD19^+^ events were gated. A subset of 20,000 CD19^+^ events for each disease and healthy group were randomly selected for normalization purposes, subsequently concatenated in a 160,000 total events fcs file and used for t-Distributed Stochastic Neighbor Embedding (tSNE) analysis (19). The following parameters were included into the t-SNE calculation: CD19-PE-Cy7-A, CD24-PE-A, CD27-APC-A, CD38-PerCP-Cy5.5-A, IgM-FITC-A, IgG-APC-H7-A, IgD-V450-A, CD21-BV605-A. t-SNE maps were generated by plotting each event by its t-SNE dimensions. Then, the Flow Cytometric Self-Organizing Map (FlowSOM) function was used to automatically identify B-cell subsets (20).

Star charts were obtained from 40 fcs files of stained samples from healthy controls and study patients. After gating B cells and normalizing each sample to 5,000 CD19^+^ events, the concatenated file of 200,000 total events was used for FlowSOM analysis. The following parameters were included into FlowSOM calculation: CD19-PE-Cy7-A, CD24-PE-A, CD27-APC-A, CD38-PerCP-Cy5.5-A, IgM-FITC-A, IgG-APC-H7-A, IgD-V450-A, CD21-BV605-A.

### Statistical Analysis

Data were summarized as medians, 25th, 75th percentiles, and range (min and max). The non-parametric Kruskal–Wallis test was used to compare more than two independent groups, whereas pairwise comparisons were evaluated by the Mann–Whitney *U*-test. A Wilcoxon matched-pairs signed rank test was performed for matched-paired data, while correlation between two non-parametric continuous variables was assessed by Spearman’s rank correlation. *P*-values < 0.05 were considered statistically significant and reported as two-sided. All statistics were carried out with the use of STATA/SE 12.0 for Windows.

## Results

### B-Cell Subsets in Study and Healthy Pediatric Cohorts

**FIGURE 1:**
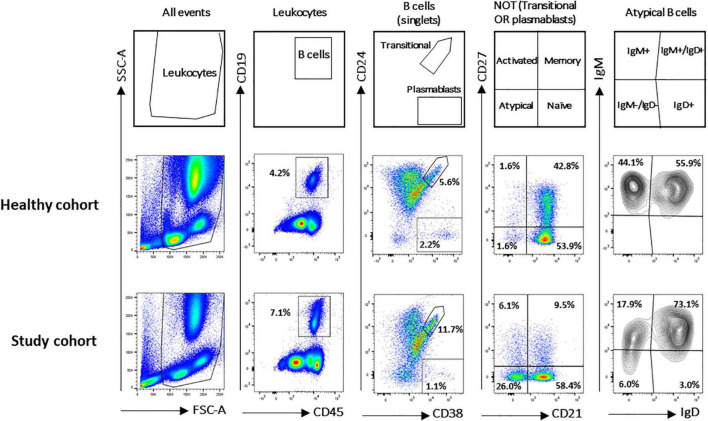
Gating strategy for B-cell subsets identification in study and healthy cohorts. B-cell subsets were identified *via* CD19, CD24, CD27, CD38, IgM, IgG, IgD, and CD21 markers. Gating strategy on two representative blood samples is shown. Names defining the different B-cell populations are shown in the empty plots.

A flow cytometry staining was performed on 1,571 and 50 fresh PB samples collected from pathological pediatric patients and healthy children, respectively. The combination of antibodies allowed the identification of several B-cell populations: transitional B cells, naïve B cells, MBCs, atBCs, actMBCs, and plasmablasts (Figure 1). The relative percentages and statistics of the B-cell subsets in the two studied cohorts are shown in Table 2. An in-depth age-related analysis of B-cell subsets was also performed comparing percentages between ten different age ranges to assess the age-related changes observed during childhood (Supplementary Figures 1A,B). Transitional and naïve B cells were shown to decrease with age, whereas MBCs significantly increased during the first 3–4 years of age. Percentages of plasmablasts, actMBCs, and atBCs were similar except for the first year of life when they resulted significantly lower.

**TABLE 2:** Statistics of the B-cell subsets on the overall flow cytometric analysis.

	Study cohort	Healthy cohort	

	***N* = 1571**	***N* = 50**	***P***
**Total B cells (% of leucocytes)**			
Median	5.0	5.1	0.519
Range	From 0.2 to 59.9	From 0.7 to 18.3	
25th percentile	3.1	4.0	
75th percentile	8.0	8.2	
**Naïve (% of B cells)**			
Median	63.4	61.7	0.857
Range	From 0.5 to 95.4	From 44.3 to 78.8	
25th percentile	54.7	57.1	
75th percentile	70.4	68.7	
**Transitional (% of B cells)**			
Median	9.8	9.0	0.737
Range	From 0.2 to 86.1	From 1.9 to 33.6	
25th percentile	6.3	7.0	
75th percentile	14.4	13.8	
**MBCs (% of B cells)**			
median	15.3	16.5	0.078
range	From 0.24 to 59.6	From 3.9 to 39.5	
25th percentile	9.9	12.9	
75th percentile	22.2	24.7	
**actMBCs (% of B cells)**			
Median	1.6	1.9	0.492
Range	From 0 to 69.3	From 0 to 5.2	
25th percentile	0.9	1.1	
75th percentile	2.7	2.8	
**Plasmablasts (% of B cells)**			
Median	2.4	2.5	0.426
Range	From 0 to 48.3	From 0.3 to 11.6	
25th percentile	1.3	1.7	
75th percentile	4.4	4.8	
**atBCs (% of B cells)**			
Median	2.4	2.3	0.430
Range	From 0.14 to 66.5	From 0.1 to 5.2	
25th percentile	1.5	1.6	
75th percentile	3.9	3.4	
≤ 5.0%	1,325 (84.3%)		
> 5.0 and ≤ 10.0%	159 (10.1%)		
>10.0 and ≤ 20.0%	54 (3.4%)		
> 20.0%	33 (2.1%)		

In the study cohort a positive correlation was observed between percentages of actMBCs and both MBC (ρ = 0.54, *P* < 0.001) and atBC (ρ = 0.61, *P* < 0.001) populations, suggesting an inter-relationship among the three types of B-cell subsets. On the contrary, there was a negative correlation between percentages of naïve B cells and MBC (ρ = –0.55, *P* < 0.001) or actMBC (ρ = –0.63, *P* < 0.001) populations.

### atBCs Features in Health and Disease: Distribution, Immunoglobulin Isotypes, and Immunophenotype

**FIGURE 2:**
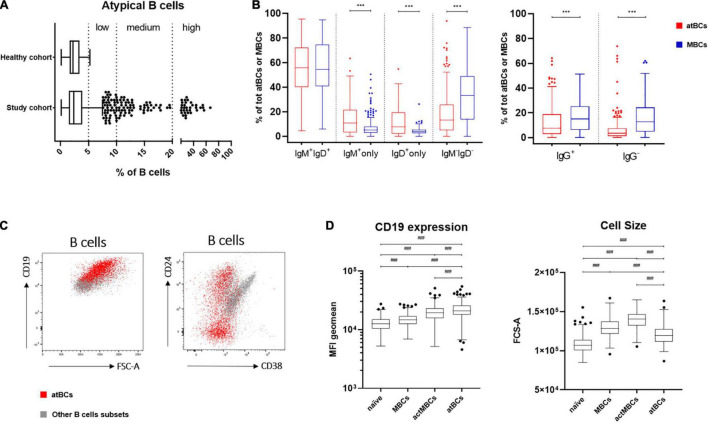
atBCs features: distribution, immunoglobulin isotypes and specific immunophenotype. **(A)** Box plots showing distribution of atBCs in the study and the healthy cohorts. Midline, lower and upper limits represent median, the first and the third quartiles, respectively. Whiskers indicate lower quartile –1.5 × IQR (interquartile range) and upper quartile + 1.5 × IQR. Outliers are shown as dots. Dotted lines separate atBCs based on percentages: ≤ 5%, > 5%, and ≤ 10% (low increase), > 10% and ≤ 20% (medium increase), > 20% (high increase). **(B)** Box plots showing the comparisons of IgM, IgD, or IgG expressing B cells between atBC and classical MBC subsets. Only samples with atBCs > 5% are represented. **(C)** FACS dot plots of a representative sample with DiGeorge syndrome showing atBCs (in red) and other B-cell subsets (in light gray) based on expression of CD24, CD38, and CD19 and FSC-A. **(D)** Comparison by box plots of CD19 MFI (mean fluorescence intensity) levels and FCS-A values among naïve, MBCs, actMBCs, and atBCs. Only samples with atBCs > 5% are represented. Statistical significances were determined using unpaired two-tailed Mann–Whitney *U*-tests or Kruskal–Wallis test with adjusted *p*-value at 0.004167, ^***^p < 0.001; ^###^*p* < 0.004167.

In the study cohort the frequency of atBCs, expressed as percentage of the B-cell population, presented a right-skewed distribution (skewness = 5.69), with 8.9% of the data points (139/1,571) appearing as outliers [> Q3 (upper quartile) + 1.5 × IQR (interquartile range)] (Figure 2A). AtBCs were also observed in the healthy cohort and the comparison with the study cohort did not show any statistically significant difference (medians: 2.4 vs. 2.3%, respectively, *P* = 0.430) (Table 2). We then focused on the wider percentage range of atBCs observed in the study cohort (between 0.14 and 66.5%) compared to the healthy cohort (between 0.1 and 5.2%), analyzing all samples with atBCs percentages greater than 5%. For this purpose, the study cohort samples were subdivided into four classes based on relative percentages of atBCs (Table 2 and Figure 2). Most of the PB samples (1,325/1,571, 84.3%) presented a percentage of atBCs ≤ 5%, whereas a low increase of atBCs (> 5% and ≤ 10%) was detected in 10.1% (159/1,571) of the samples. A medium increase of atBCs percentages (> 10% and ≤ 20%) was observed in 3.4% (54/1,571) and a high increase (> 20%) was recorded in 2.1% (33/1,571) of the study cohort samples.

Previous immunoglobulins expression analysis on atBCs of healthy and non-healthy adults demonstrated the presence of switched and unswitched B cells (2, 21), whereas very little is known about immunoglobulins expression distribution in children. With this aim, we selected samples with relative atBCs percentages > 5% (246/1,571), we analyzed the expressions of IgM and IgD in this B-cell subset and compared them with MBC population. Four subpopulations with different expression of the two immunoglobulins were discriminated: IgM^+^IgD^+^, IgM^+^-only, IgD^+^-only, and IgM^–^IgD^–^ (Figure 2B). IgM^+^IgD^+^ B cells were the most abundant population among atBCs, with a median frequency of 55.8%. No differences in IgM^+^IgD^+^ B cells frequency were observed between atBCs and MBCs (55.8 vs. 54.3%, *P* = 0.913). On the contrary, IgM^+^-only and IgD^+^-only B cells were significantly higher in atBCs compared to MBCs (10.7 vs. 5.1%, *P* < 0.001 and 7.6 vs. 4.0%, *P* < 0.001, respectively). Moreover, a significant lower frequency of IgM^–^IgD^–^ switched B cells was detected in atBCs compared to the MBCs (13.3 vs. 33.2%, *P* < 0.001). Furthermore, the expression of IgG was evaluated on IgM^–^IgD^–^ atBC and IgM^–^IgD^–^ MBC subsets. IgG^+^ atBCs were higher than IgG^–^ atBCs within the IgM^–^ IgD^–^ B-cell subpopulation (7.7 and 3.5%, respectively), although significantly lower than IgG^+^ MBCs (7.7 vs. 15.1%, *P* < 0.001) (Figure 2B). Interestingly, IgM^+^-only atBCs expressed the immunoglobulin isotype with a lower density (dim expression) than MBCs (MFI, 2,185 vs. 6,634, *P* < 0.001). On the contrary, IgD immunoglobulin was expressed by IgD^+^-only atBCs at a higher level compared to MBCs (MFI, 8,518 vs. 2,781, *P* < 0.001) (Supplementary Figure 2A).

To complete the in-depth immunophenotyping of atBCs, the expression of CD38, CD24, and CD19 was then investigated. Due to the extremely low levels or absence of CD38 expression on atBCs, the use of this cell surface marker allowed a clearer discrimination of this specific B-cell subset. Conversely, CD24 was present in a more heterogeneous way on atBCs, with a median frequency of 27% (range from 0.6 to 93.7%) (Figure 2C). Immunoglobulin isotypes distribution of CD24^+^ atBCs reflected the overall atBCs expression frequency: IgM^+^IgD^+^ B cells were the most abundant population (median frequency 68.8%) (Supplementary Figure 2B). Of note, CD19 expression level was significantly higher in atBCs than in MBCs, actMBCs, and naïve B cells (Figure 2D). The cell size across the different B-cell subsets was also analyzed. The atBC population showed a significantly higher FCS-A (forward scatter, Area) than naïve B cells but a lower FCS-A value than both MBCs and actMBCs (Figure 2D).

### Correlation Between atBCs and Clinical Features

**FIGURE 3:**
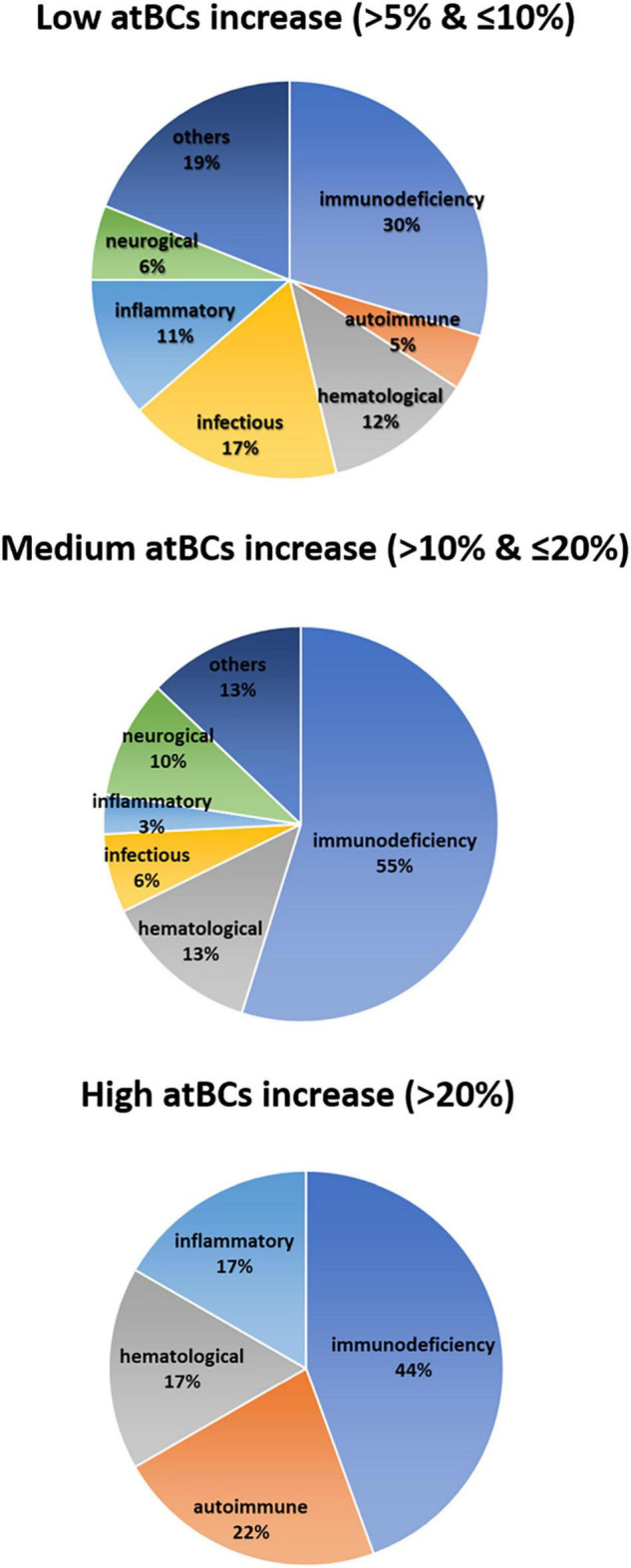
Correlation between atBCs frequencies and pediatric disorders. Pie charts showing the frequency of observed pediatric diseases within the three groups of atBCs increase (low, medium, and high).

In order to assess the association between the increase of atBCs in PB samples and pediatric diseases, we performed correlation analyses of the flow cytometric findings with children’s diagnosis or main clinical features. First, patients with atBCs > 5% (181/1,180, 15.3%) were selected and categorized into seven types of disorders, based on the main clinical findings, diagnosis, or presumptive diagnosis. For patients with more than one flow cytometric evaluation during the study time, only the first assessment was included in the analysis. As expected, patients re-evaluated after at least 1 month, did not show any significant change in atBCs percentages [9.4 (T_0_) vs. 9.7% (T_1_), *P* = 0.910] (Supplementary Figure 3). Subsequently, the seven clinical categories were associated to a low (> 5% and ≤ 10%), medium (> 10% and ≤ 20%), and high (> 20%) increase of atBCs. Among the selected patients, 132 (11.2%), 31 (2.6%), and 18 (1.5%) children showed a low, medium, and high increase of atBCs, respectively. As shown in Figure 3, all clinical disorders were represented in the group with a low increase of atBCs, with a slight prevalence of the immunodeficiencies (30%). Immunodeficiencies, as a main clinical finding, diagnosis, or presumptive diagnosis, accounted for approximately half of the clinical conditions in children with both medium and high frequencies of the atBC population (55 and 44%, respectively). Autoimmune and inflammatory diseases were more frequently associated with samples with a high increase of atBCs (22 and 17%, respectively). Hematological patients were associated with comparable frequency to all three atBC groups (12% in the low, 13% in the medium, and 17% in the high atBC groups). Infectious diseases, neurological, and other disorders were not observed in the group with the highest percentage increase of atBCs (Figure 3).

The prevalence of the specific disorders in the study cohort, associated to a low (> 5 and ≤ 10%), medium (> 10 and ≤ 20%), and high (> 20%) increase of atBCs are reported in the Supplementary Table 1. The most frequently observed immunodeficiencies were: DiGeorge syndrome (15/64, 23%), IgA deficiency (11/64, 17.2%), combined immunodeficiencies (CID), and severe combined immunodeficiencies (SCID) (11/64, 17.2%), Ataxia-telangiectasia (4/64, 6.3%), common variable immunodeficiencies (CVID) (4/64, 6.3%), Wiskott-Aldrich syndrome (3/64, 4.7%), acquired immune deficiency syndrome (AIDS) (2/64, 3.1%), and primary hemophagocytic lymphohistiocytosis (HLH) (2/64, 3.1%). Three patients with SCID were evaluated after hematopoietic stem-cell transplantation (HSCT).

**FIGURE 4:**
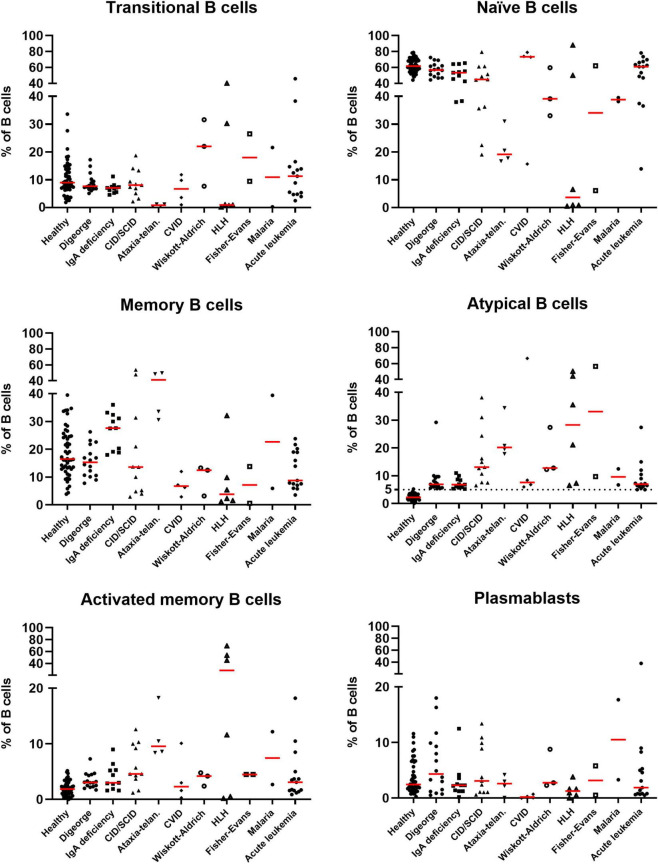
Identification of B-cell subsets in selected pediatric disorders. Scatter dot plots indicating the percentages of B-cell subsets in selected diseases in patients with increased atBCs (> 5%). Bars in red indicate medians.

When comparing relative percentages of atBCs in patients suffering from immunodeficiencies, we observed higher median values in patients with ataxia-telangiectasia and CID/SCID. However, the highest relative percentage of atBCs was detected in a patient with CVID (Figure 4). In contrast, DiGeorge Syndrome and IgA deficiency patients had the lowest median percentages of atBCs. No significant differences were observed neither for immunoglobulin isotypes nor for CD24 expression on atBCs across all immunodeficiencies (Supplementary Figure 4).

Among the autoimmune diseases with increased percentages of atBCs, we were able to observe Fisher-Evans syndrome (2/11, 18.2%), immune thrombocytopenia (2/11, 18.2%), ANCA glomerulonephritis, HLH associated with connective tissue disease, and autoimmune hemolytic anemia (all 1/11, 9%) (Supplementary Table 1).

As part of the inflammatory diseases, secondary HLH (3/18, 16.7%), Crohn’s disease (3/18, 16.7%), juvenile idiopathic arthritis (3/18, 16.7%), A20 haploinsufficiency (2/18, 11.1%) were the most frequently observed among patients with atBCs > 5%. Patients with secondary HLH were only observed within the high atBCs increase group (> 20%). Most of the inflammatory diseases were detected in the group with the lowest increase of atBCs while in the medium atBCs increase group we observed only one patient with A20 haploinsufficiency (Supplementary Table 1).

Among the hematological patients with atBCs > 5%, 14/23 (60.9%) children suffered from acute lymphoblastic or myeloid leukemia, 2/23 (8.7) from non-Hodgkin lymphoma, 2/23 (8.7%) from Fanconi anemia, and 2/23 (8.7%) from iron-deficiency anemia. Children with acute lymphoblastic or myeloid leukemia (11/14, 78.6%) and the two patients with Fanconi anemia were evaluated after HSCT. Hematological patients were present in all three atBC groups with a comparable frequency (Supplementary Table 1).

The majority of patients with infectious diseases had a modest increase of atBCs (> 5% and ≤ 10%). In most cases, they had recurrent infections not associated to previous disorders (15/25, 60%). Pulmonary tuberculosis (3/25, 12%) and Malaria (2/25, 8%) were also found to expand the atBC subset (Supplementary Table 1).

In the context of neurological diseases, early infantile epileptic encephalopathies (EIEE) (6/11, 54.5%) and cerebellar ataxia (2/11, 18.2%) were the most frequent in patients with atBCs > 5%. No patient with neurological disorders was found in the high increase group, whereas three cases (West syndrome, X linked lissencephaly, GABRA1/EIEE-19) were observed in the group with a medium increase of atBCs (Supplementary Table 1).

When focusing on all other diseases in our study cohort, cardiovascular diseases (9/29, 30%), genetic disorders (5/29, 16.7%), metabolic disorders (2/29, 6.7%), and carcinoma (2/29, 6.7%) were the most frequent in patients with atBCs between 5 and 20%. One patient with hypoplastic left heart syndrome, one with polycystic kidney disease and kidney failure and another patient who underwent surgical splenectomy showed a medium increase of atBCs percentages (Supplementary Table 1).

### High-Dimensional Data Analysis

**FIGURE 5:**
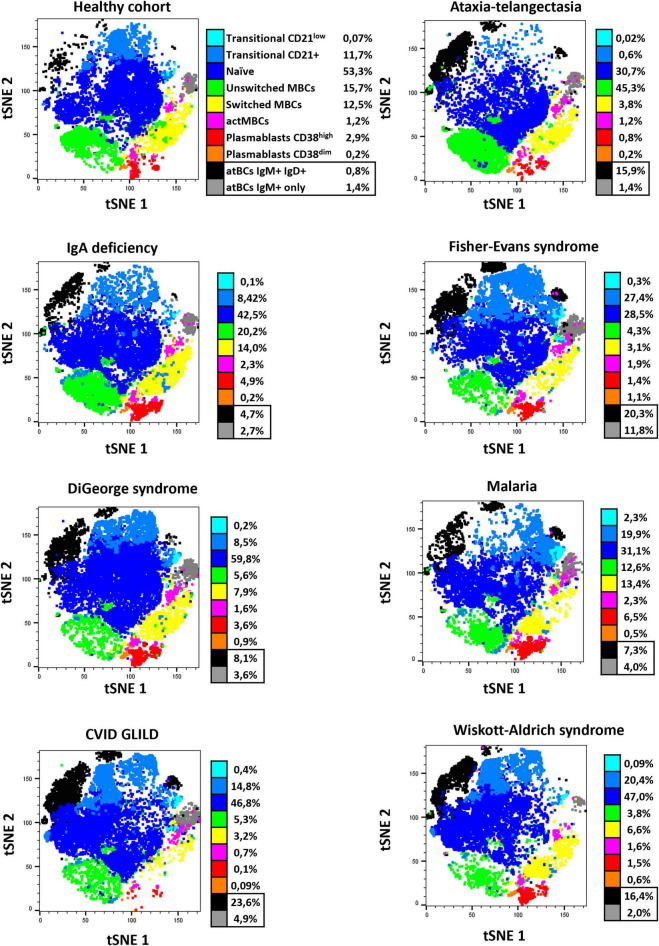
tSNE B-cell subset characterization of representative healthy pediatric individuals and study patients. Seven observed diseases in patients with atBCs increase [ataxia-telangiectasia, *n* = 2; IgA deficiency, *n* = 2; Fisher-Evans syndrome, *n* = 2; DiGeorge syndrome, *n* = 2; Malaria, *n* = 2; CVID granulomatous-lymphocytic interstitial lung disease (GLILD), *n* = 3; Wiskott-Aldrich syndrome, *n* = 2] and healthy controls (*n* = 3) were selected for a reduced dimensionality analysis, by using merged t-stochastic neighbor embedding (tSNE). Flow cytometric Self-Organizing Map (FlowSOM) was used to automatically identify B-cell subsets in each disease.

In order to appreciate high-dimensional similarities of cells and to visualize the proportions of the atBC population in patients with different diagnosis, data from B-cell phenotype have been represented as a t-distributed Stochastic Neighbor Embedding (tSNE) plot after data integration and batch correction. Separated tSNE plots for samples from representative diseases and healthy children are shown in Figure 5. Ten clear immunophenotypic clusters were automatically identified and recognized using Flow cytometric Self-Organizing Map (FlowSOM). The two identified subsets of atBCs (IgM^+^IgD^+^ and IgM^+^-only) were visually and quantitatively increased in representative diseases compared to the healthy cohort. Differences in other B-cell subsets were easily evaluable (Figure 5).

**FIGURE 6:**
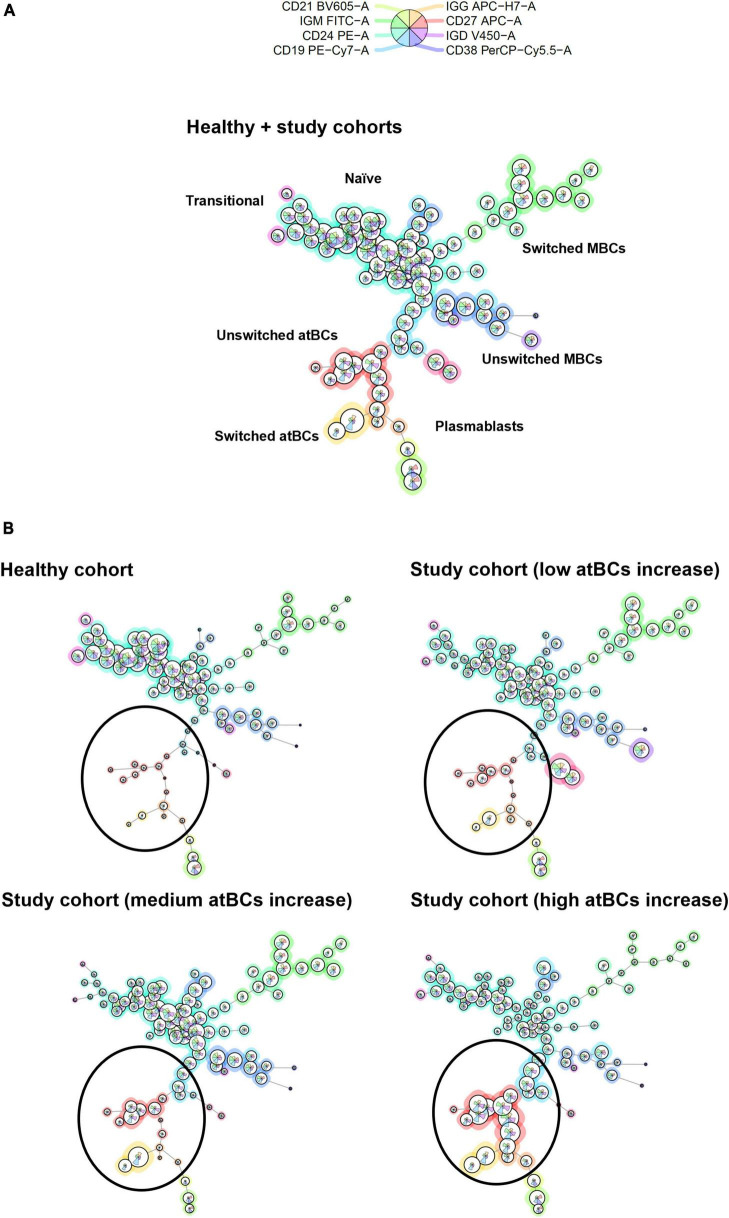
B-cell subsets clustering by FlowSOM star charts. **(A)** Thirty patients from the study cohort (low atBCs increase, *n* = 10; medium atBCs increase, *n* = 10; high atBCs increase, *n* = 10) and 10 healthy controls were selected for a graph-based clustering analysis, using flow cytometric Self-Organizing Map (FlowSOM). **(B)** Star chart of all 40 patients. The background coloring of nodes indicates the clustering. Definitions of the different B-cell subsets are shown for each clustering. Comparison by FlowSOM star charts of healthy group and the three classes of patients with increased atBCs (low, medium, and high). Black circles highlight atBCs clustering.

A star chart visualization by FlowSOM was applied to the immunophenotypic analysis of selected patients from both study and healthy cohorts in order to investigate cluster relationships among all B-cell subsets (Figure 6A). As shown in Figure 6B, atBCs established a well-defined and divergent cluster compared to MBCs. Moreover, a high increase of atBCs percentages (> 20%) corresponded to a marked reduction of unswitched and, more in particular, switched MBCs.

## Discussion

In this study, we performed an extensive flow cytometric evaluation of atBCs in blood samples of a comprehensive pediatric population affected by a variety of disorders (0–18 years-old). Due to the heterogeneity in the definition of atBCs, namely tissue-like memory B cells, atypical memory B cells, CD11c^+^ B cells, T-bet^+^ memory B cells, double-negative CD27^–^ IgD^–^ B cells, and CD21^–/low^ B cells, we first asked whether our routinely applied immunophenotypic panel was suitable to correctly characterize this B-cell subset in different pathologies. The first description of a unique B-cell subpopulation (CD20*^hi^*/CD27*^low^*/CD21*^low^*) was reported by Moir S et al. (1) in the peripheral blood of HIV-viremic individuals and termed tissue-like memory B cells, similar to B cells in tonsillar tissues expressing the inhibitory receptor Fc-receptor-like-4 (FCRL4) (7). These B cells lacked the complement receptor CD21 and the tumor necrosis family receptor CD27, they also expressed several inhibitory receptors and were refractory to BCR stimulation alone but responsive with concomitant TLR-9 stimulation *in vitro*. In a later study, a high level of T-bet expression was demonstrated on these B cells in HIV + subjects (22). The overlapping phenotype CD21*^low^*, CD27*^low^*, T bet^+^, CD11c^+^ was confirmed on this B-cell subset in other pathologies, such as SLE (23, 24), Crohn’s disease (25), Sjögren’s syndrome (16), rheumatoid arthritis (RA) (14), CVID (11, 14, 26), and hepatitis B and C (27, 28), malaria (29, 30), and tuberculosis (31). Although different immunophenotypic definitions of the atypical B cells still exist, in the most recent publications, there is a general consensus for a common origin of this specific B-cell subset (4, 5). As a result, the simultaneous presence of CD19, CD27, and CD21 in our panel has been essential for the identification of atBCs, whereas CD24 and CD38 have been useful to exclude transitional B cells (CD24^+^, CD38^++^, CD21^±^) and plasmablasts (CD24^–^, CD38^++^, CD27^++^, CD21^–^). The staining and the gating strategies proved to be optimal to compare relative percentages of atBCs in different pathologies, as previously reported (21, 30, 32–35). More recently, a study based on single-cell RNA sequencing showed that CD21^–^ CD27^–^ B cells represent “true” atBCs, defined as an alternative lineage of B cells implicated in conventional immune responses to vaccination and infections in humans (8). The hypothesis of an alternative B-cell lineage is corroborated by our study on children since the immunophenotype of atBCs clustered separately and specularly compared to classical MBCs by using a new data visualization technique (FlowSOM).

In Thorarinsdottir K et al. (2) demonstrated that CD21^–/low^ B cells were memory cells since they displayed markers indicating previous activation, such as CD95 and CD62L, similar to classical memory B cells and also lacked expression of the ABCB1 transporter, normally present on naïve B cells. Furthermore, the authors detected a low percentage of CD21^–/low^ B cells in the PB of healthy adults. An expansion of these cells was then observed in disease settings or after vaccination with live virus vaccines, including influenza, vaccinia, and yellow fever viruses (36, 37). Therefore, the assessment of peripheral blood atBC levels might be used as an indicator of healthy conditions or of the presence of other disorders. Normal percentage of CD21^–/low^ B cells in healthy adult females is approximately 5% (median 4.6%; 25th and 75th percentiles: 3.5–6.2%) of B cells (2). In CVID EUROClass trial (12) normal CD21*^low^* B cells ranged from 1.1 to 6.9% of B cells. Morbach, H et al. (38) provided reference values of different B-cell subpopulations in healthy individuals. CD21*^low^* CD38*^low^* B-cells were included in the analysis and percentages relative to total B cells ranging from 0.3 to 5.2% (25th and 75th percentiles, respectively) in 64 evaluated individuals (age range from 0 to 50 years-old). In our study, the observed percentage of atBCs in 50 healthy children ranged from 0.1 to 5.2% of total B cells.

Besides the low expression of CD21 and CD27, a reduced or absent expression of CD38 on atBCs was noticed since the first description of atBCs in tonsillar tissues (7). In fact, the low expression of CD38 on this B-cell subset was also described in the peripheral blood of patients with CVID (11), RA (39), SLE (15), and in healthy individuals (2). Consistent with previous works, we observed a low expression of CD38 on all analyzed CD21^–^ CD27^–^ atBCs, highlighting the critical role of this marker in the immunophenotypic characterization of this B-cell subset. Conversely, the expression of CD24 on atBCs in our study cohort appeared heterogeneous. However, the CD24^+^ atBC population reflected the IgM and IgD expression on atBCs and was not associated to specific pathologies. Vlkovà M et al., (40) described two discrete subpopulations of CD21^–^ CD27^–^ CD38^–^ B cells based on the expression of CD24 in CVID patients. Both populations showed a markedly increased proliferation in patients with CD21^–^ CD27^–^ CD38^–^ B cells > 10%, as compared to healthy controls, suggesting a stunted development rather than an increased bone marrow output. Furthermore, authors observed a higher expression of CD19 on atBCs, as previously reported (11). Moreover, in CVID patients, CD21*^low^* B cells cellular size is significantly larger than that of naïve B cells (26). The higher expression of CD19 and the larger dimension of atBCs, compared to naïve B cells, were confirmed in our extensive analysis, suggesting that these are common features of atBCs in different diseases.

Although the presence of two subsets of CD21*^low^* B cells, based on IgM expression (IgM*^low^* and IgM*^hi^*), was described in CVID patients (11), a more detailed study on immunoglobulin isotype expression has been more recently reported (2). The study described approximately 50% of IgM^+^IgD^+^ in CD21*^low^* B cells of healthy individuals and 15% of IgM^+^ IgD*^dim^*. Moreover, approximately 15% of CD21*^low^* B cells were IgG^+^ and half as many were IgA^+^. Our findings on immunoglobulin isotypes distribution of atBCs are consistent with this data. We observed that both IgM^+^ IgD^+^ and IgM^+^-only were the most abundant subsets in atBCs. In addition, we detected, at lower frequency (7.6%), a subpopulation IgD^+^-only. IgG^+^ atBCs were predominant in IgM^–^ IgD^–^ subpopulation and despite the absence of staining, we hypothesize the remaining part being IgA^+^, as we have previously reported (41).

Our extensive analysis confirmed, on a pediatric cohort, that the increase of CD21^–^ CD27^–^ B cells percentage is mainly correlated to immunodeficiencies, autoimmune, inflammatory, and hematological diseases, as previously reported on adult populations. The CD21*^low^* B-cell subset has been widely investigated for over 10 years with most of the studies conducted mainly on adult CVID patients. In these patients, CD21*^low^* B cells were associated to splenomegaly and autoimmune cytopenia (11). In a different study, CVID patients with over 20% of CD21*^low^* B cells were shown to be more prone to develop autoimmunity and splenomegaly. Subsequently, the EUROClass CVID classification correlated the increase of CD21*^low^* B cells also to granulomatous disease (12). Furthermore, elevated frequency of CD21*^low^* B cells were previously reported in patients with ataxia-telangiectasia (42–44) and Wiskott-Aldrich syndrome (45), as we have also observed in our pediatric cohort. In addition, we reported a high increase of atBCs in children with CID and SCID, and a low increase in patients with DiGeorge syndrome and IgA deficiency, as previously described (46–48). In two children with HIV + /AIDS an increase > 10% of atBCs was detected, corroborating results obtained in other studies (1).

A strong association between elevated CD21*^low^* and autoimmune diseases in adults, such as SLE, RA, multiple sclerosis, and Sjögren’s syndrome, is well-documented (14, 16, 23, 24, 49). In our pediatric cohort, patients with SLE and RA presented an expansion of atBCs, as well as children with Fisher-Evans syndrome, immune thrombocytopenia (ITP), and autoimmune hemolytic anemia (AIHA), in which increased CD21*^low^* CD11c^+^ B cells were previously observed (50, 51).

Most of the recent data on atBCs come from studies on malaria. It has been demonstrated that atypical memory B cells in children and adults in endemic areas, can represent over 50% of all circulating B cells (30). We could observe two patients with malaria within the low and medium atBCs increase groups and one patient with Leishmaniasis in the medium increase group, confirming the possible involvement of atBCs in response to parasitic infections (10, 52). In studies focusing on different infections, atBCs have shown the ability to produce isotype switched antibodies, contributing to the protective response against the infectious agent, therefore whether atBCs are dysfunctional cells still remains controversial (53–55). More recently, CD21^–^ CD27^–^ atBCs in 14 malaria-exposed children were described as switched B cells (21). The herein presented malaria cases were too few to confirm previous reports on the subject.

In pediatric patients undergoing HSCT, chronic graft-*vs*-host disease (cGVHD) is associated with changes in the B-cell compartment, including a lowering in frequencies of CD27^+^ MBCs and an increase in frequencies of circulating CD21*^low^* B-cells. However, resolution of cGVHD correlates with the expansion of CD27^+^ MBCs and the normalization of CD21*^low^* B-cell frequencies (56). In our cohort, 23 patients with malignant and non-malignant hematologic disorders showed different levels of atBCs increase. Although we had no available information about clinical complications, 13 out of 23 patients underwent HSCT.

Importantly, we observed an increase of atBCs in children with neurological, cardiovascular, genetic, and metabolic disease; therefore, further investigations will be performed in order to understand if this increase is due to primary diseases or comorbidities.

In conclusion, we demonstrated the expansion of atBCs in several pediatric pathologies by flow-cytometric assessment of B-cell subsets. Specifically, the highest atBCs increase was correlated with immunodeficiency, autoimmune, inflammatory, and hematological disorders. In our study cohort, atBCs were phenotypically homogenous across the different pathologies and had characteristics of unswitched B cells, with only a minor fraction of switched atBCs observed. Combined with the common approaches already in use, the detailed analysis of atBCs frequencies and phenotype herein presented could help in the identification or diagnosis of specific disorders. Data presented in this preliminary extensive study will be the basis for further investigations focused on single observed pathologies.

## Data Availability Statement

The original contributions presented in the study are included in the article/Supplementary Material, further inquiries can be directed to the corresponding author/s.

## Ethics Statement

Ethical review and approval was not required for the study on human participants in accordance with the local legislation and institutional requirements. Written informed consent from the participants’ legal guardian/next of kin was not required to participate in this study in accordance with the national legislation and the institutional requirements.

## Author Contributions

FC, PP, CC, and RC: conceptualization, data analysis, and validation. FC, ST, PP, CC, MM, and RC: investigation and methodology. FC, PP, CC, and MM: data curation. FC: statistical analysis. FC and ST: visualization and writing—original draft. FC, ST, PP, and RC: writing—review and editing. CP and RC: supervision, funding acquisition, and project administration. All authors contributed to the article and approved the submitted version.

## Conflict of Interest

The authors declare that the research was conducted in the absence of any commercial or financial relationships that could be construed as a potential conflict of interest.

## Publisher’s Note

All claims expressed in this article are solely those of the authors and do not necessarily represent those of their affiliated organizations, or those of the publisher, the editors and the reviewers. Any product that may be evaluated in this article, or claim that may be made by its manufacturer, is not guaranteed or endorsed by the publisher.

## Supplementary Material

Supplementary Figure 1 Age-related B-cell subset percentages in study and healthy cohorts. **(A)** Box plots showing B-cell subsets (transitional, naïve, memory, activated memory, atypical B cells, and plasmablasts) in the study cohort divided in ten different age ranges (< 1 y: *n* = 126; 1–2 y: *n* = 246; 3–4 y: *n* = 226; 5–6 y: *n* = 198; 7–8 y: *n* = 156; 9–10 y: *n* = 160; 11–12 y: *n* = 132; 13–14 y: *n* = 128; 15–16 y: *n* = 130; 17–18 y: *n* = 68). Boxes are shown with median, lower and upper limits (first and third quartiles, respectively). Whiskers indicate lower quartile −1.5 × IQR (interquartile range) and upper quartile + 1.5 × IQR. Outliers are shown as dots. **(B)** Matrices for statistical test significance representation among age ranges for each B-cell subset in the study cohort. Each matrix reports significance for two distinct B-cell subsets. Statistical significance was determined using Kruskal–Wallis test with adjusted *p*-value at 0.000556.

Supplementary Figure 2 atBCs features in the study cohort. **(A)** Box plot comparison of IgM or IgD Mean Fluorescence Intensity (MFI) values, within IgM^+^-only or IgD^+^-only subpopulations, between atBC and MBC subsets. Only samples with atBCs > 5% are represented. **(B)** Distribution by box plots of IgM and IgD expressing B cells within the CD24^+^ atBC subset in those samples in the study cohort with atBCs > 5%. Statistical significances were determined using unpaired two-tailed Mann–Whitney *U*-tests or Kruskal–Wallis test with adjusted *p*-value at 0.004167, ^***^*p* < 0.001; ^###^*p* < 0.004167.

Supplementary Figure 3 atBCs percentages at first (T0) and second (T1) flow cytometric assessment. Before-after graph showing changes of atBCs percentages at two sampling time points. Statistical significance was determined using Wilcoxon matched-pairs signed rank test.

Supplementary Figure 4 Identification of immunoglobulin isotypes in selected pediatric disorders. Scatter dot plots indicating the percentages of CD24^+^, IgM^+^ IgD^+^, IgM^+^-only, IgD^+^-only, IgM^–^ IgD^–^, IgG^+^ atBCs in selected disorders in patients with increased atBCs (>5%). Red bars indicate medians.

Supplementary Table 1 atBCs increase groups and associated diseases. Tables showing specific diseases and number of observations in each group of increased atBCs (low, medium, and high).
