# Supplementary material for: CD21– CD27– Atypical B Cells in a Pediatric Cohort Study: An Extensive Single Center Flow Cytometric Analysis
Source: Front Pediatr. 2022 Jun 3;10:822400. doi: 10.3389/fped.2022.822400 (PMC9204099; doi:10.3389/fped.2022.822400)
Supplement: Supplementary Figure 1 — Age-related B-cell subset percentages in study and healthy cohorts. (A) Box plots showing B-cell subsets (transitional, naïve, memory, activated memory, atypical B cells, and plasmablasts) in the study cohort divided in ten different age ranges (< 1 y: n = 126; 1–2 y: n = 246; 3–4 y: n = 226; 5–6 y: n = 198; 7–8 y: n = 156; 9–10 y: n = 160; 11–12 y: n = 132; 13–14 y: n = 128; 15–16 y: n = 130; 17–18 y: n = 68). Boxes are shown with median, lower and upper limits (first and third quartiles, respectively). Whiskers indicate lower quartile −1.5 × IQR (interquartile range) and upper quartile + 1.5 × IQR. Outliers are shown as dots. (B) Matrices for statistical test significance representation among age ranges for each B-cell subset in the study cohort. Each matrix reports significance for two distinct B-cell subsets. Statistical significance was determined using Kruskal–Wallis test with adjusted p-value at 0.000556. [file Data_Sheet_1.pdf]

1.A

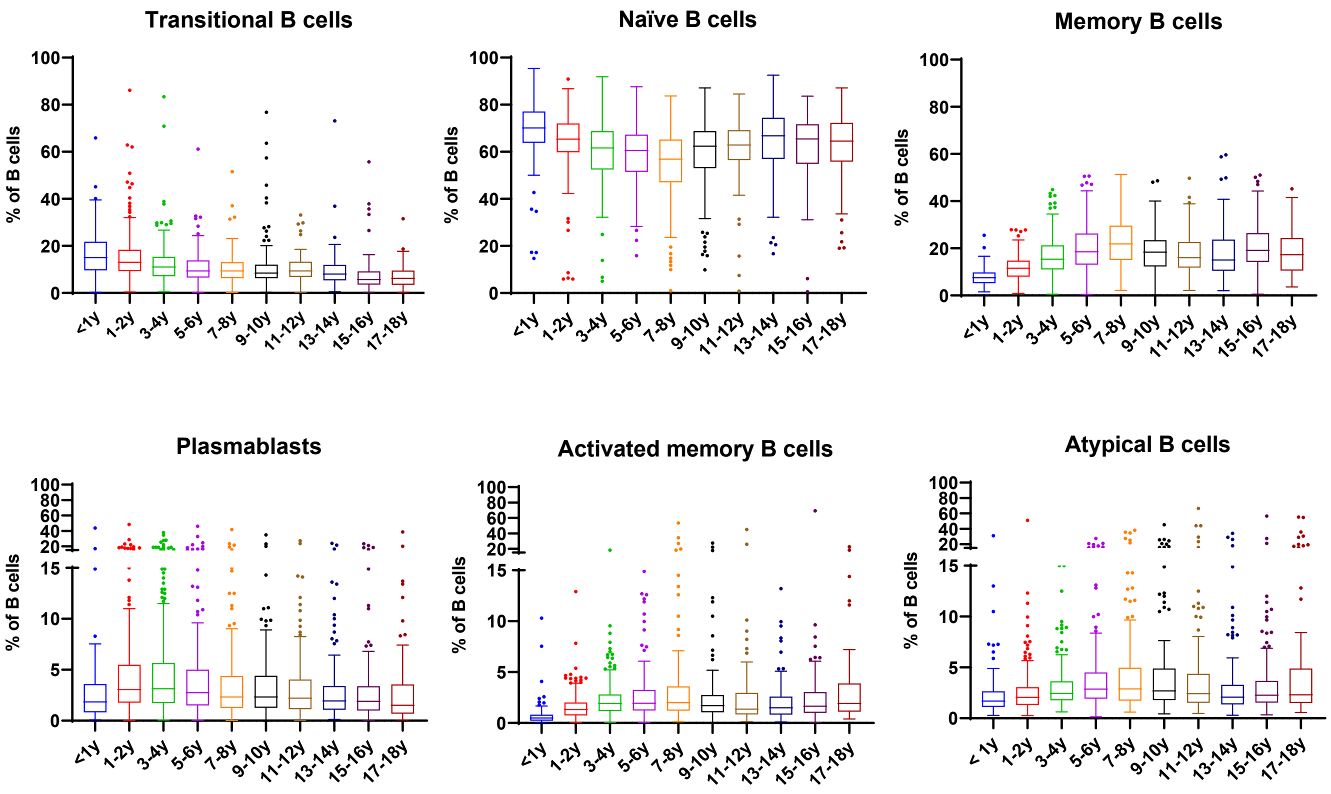

1.B

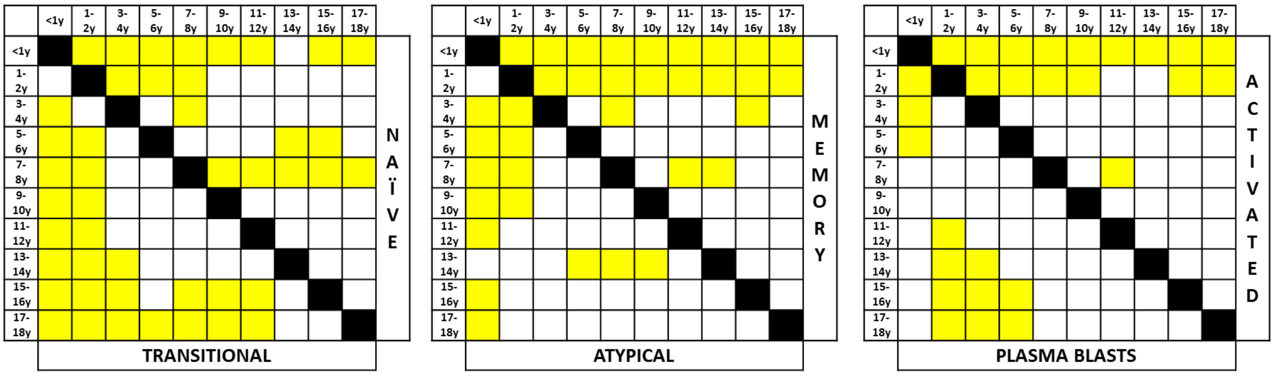

Kruskal–Wallis test: adjusted p-value for significance is 0.000556

$P < 0.000556$

$P > 0.000556$

2.A

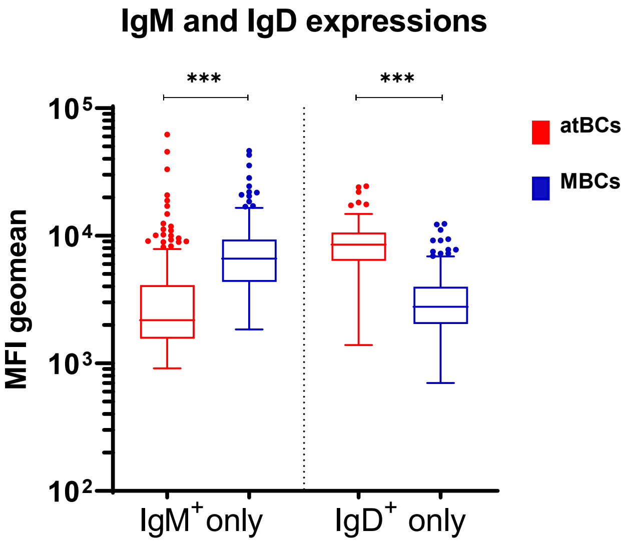
IgM and IgD expressions
***
***
10^5
10^4
10^3
10^2
MFI geomean
atBCs
MBCs
IgM+only
IgD+ only

2.B

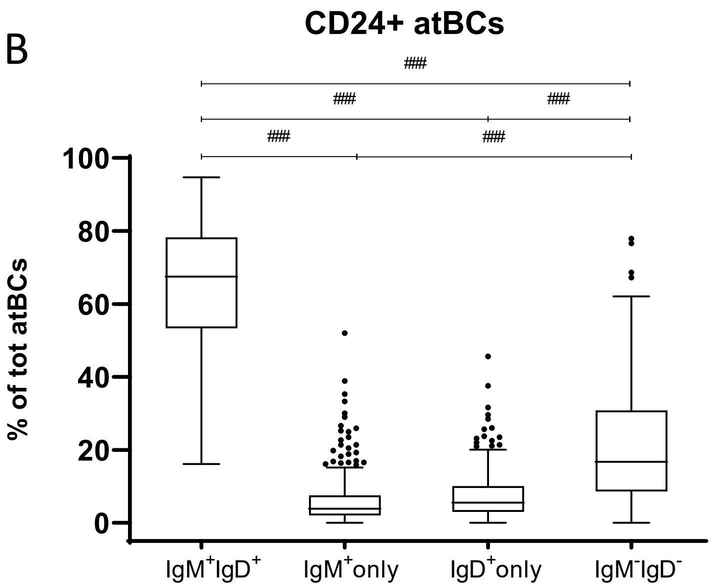
CD24+ atBCs
###
###
###
###
###
100
80
60
40
20
0
% of tot atBCs
IgM+IgD+
IgM+only
IgD+only
IgM-IgD-

3.

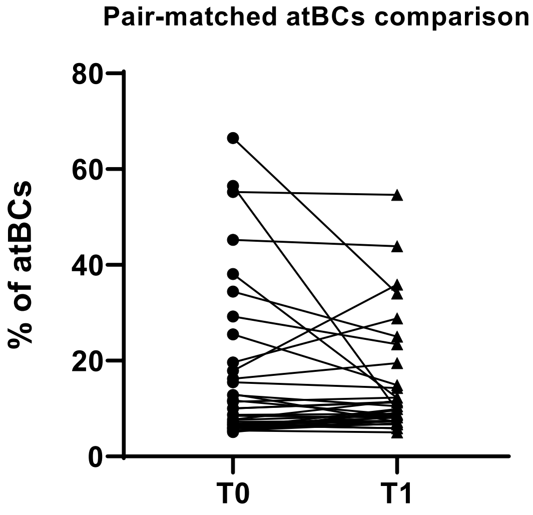
Pair-matched atBCs comparison
80
60
40
20
0
% of atBCs
T0
T1

4.

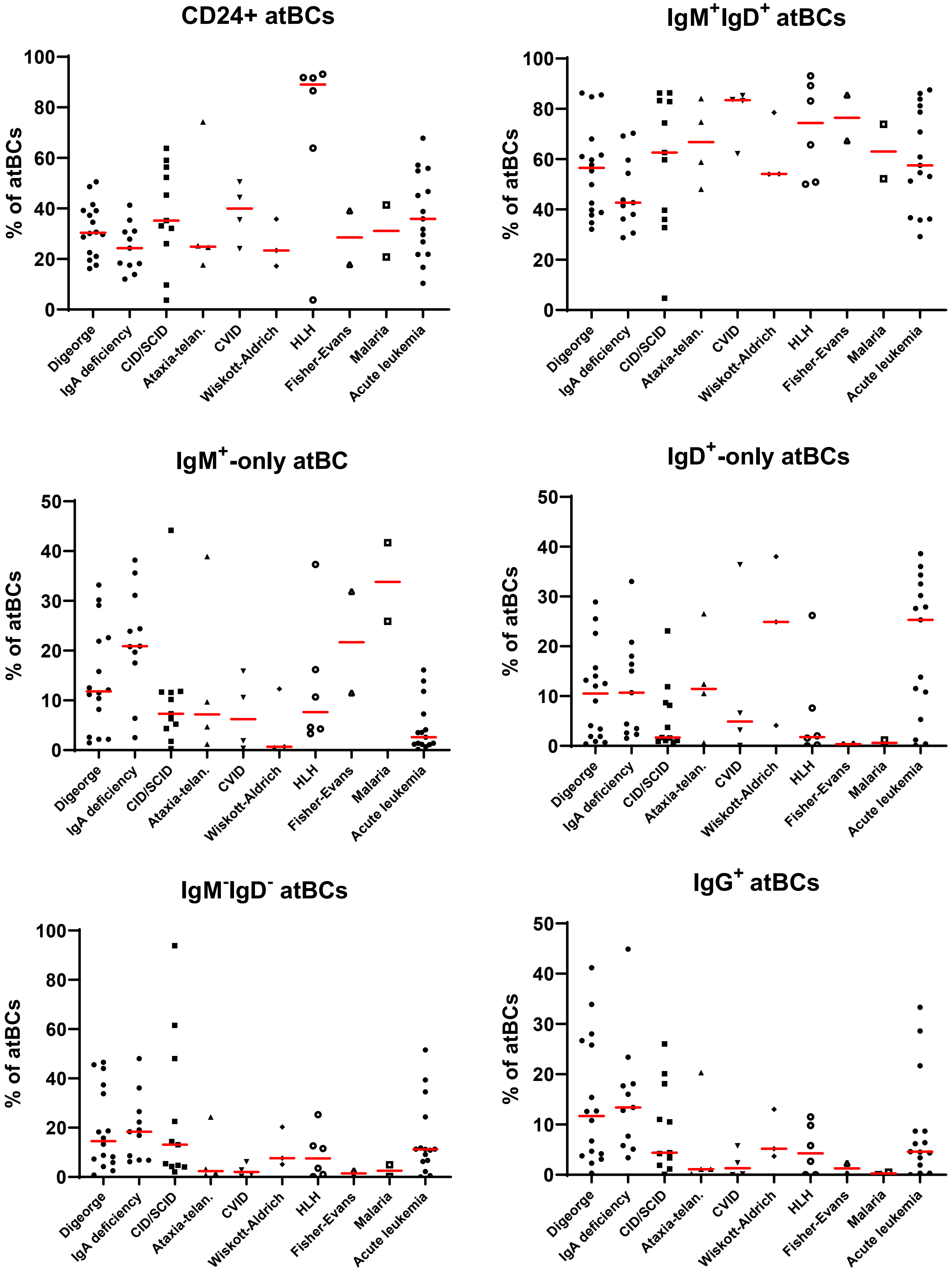
CD24+ atBCs
IgM+IgD+ atBCs
IgM+-only atBC
IgD+-only atBCs
IgM-IgD- atBCs
IgG+ atBCs
% of atBCs
Digeorge
IgA deficiency
CID/SCID
Ataxia-telan.
CVID
Wiskott-Aldrich
HLH
Fisher-Evans
Malaria
Acute leukemia

Table 1.

Low atBCs increase (>5% & ≤10%)	
**Immunodeficiency disorders**	N = 39
DiGeorge syndrome	14
IgA deficiency	10
CID/SCID	3
CVID	3
Primary HLH	2
Vici syndrome	2
Others	5
**Autoimmune diseases**	N = 6
Immune thrombocytopenia	2
Fisher-Evans syndrome	1
LES	1
Autoimmune hepatitis	1
Rheumatoid arthritis	1
**Infammatory diseases**	N = 15
Crohn's diseases	3
Juvenile idiopathic arthritis	3
Vasculitis	2
Haploinsufficiency of A20 gene	1
Others	6
**Infectious diseases**	N = 23
Recurrent infections	15
Pulmonary tuberculosis	3
Malaria	1
Others	4
**Neurological diseases**	N = 8
Early infantile epileptic encephalopathies	7
Cerebellar ataxia	1
**Hematological diseases**	N = 16
Acute lymphoblastic or myeloid leukemia	8
Fanconi anemia	2
Iron-deficiency anemia	2
Non-Hodgkin lymphoma	1
Others	3
**Other diseases**	N = 25
Cardiovascular diseases	9
Genetic disorders	5
Metabolic disorders	2
Carcinoma	2
Others	7

Medium atBCs increase (>10% & ≤20%)	
**Immunodeficiency disorders**	N = 17
CID/SCID	5
Ataxia-telangectasia	2
Wiskott-Aldrich syndrome	2
AIDS	2
IgA deficiency	1
Others	5
**Infammatory diseases**	N = 1
Haploinsufficiency of A20 gene	1
**Infectious diseases**	N = 2
Malaria	1
Leishmania	1
**Neurological diseases**	N = 3
Early infantile epileptic encephalopathies	1
West syndrome	1
X linked lissencephaly	1
**Hematological diseases**	N = 4
Acute lymphoblastic or myeloid leukemia	3
Myelodysplastic syndrome	1
**Other diseases**	N = 4
Genetic disorders	1
Hypoplastic left heart syndrome	1
Polycystic kidney disease	1
Surgical splenectomy	1

High atBCs increase (>20%)	
**Immunodeficiency disorders**	N = 8
CID/SCID	3
Ataxia-telangectasia	2
DiGeorge syndrome	1
CVID	1
Wiskott-Aldrich syndrome	1
**Autoimmune diseases**	N = 4
Fisher-Evans syndrome	1
HLH with connective tissue diseases	1
Autoimmune hemolytic anemia	1
ANCA glomerulonephritis	1
**Infammatory diseases**	N = 3
Secondary HLH	3
**Hematological diseases**	N = 3
Acute lymphoblastic or myeloid leukemia	2
Non-Hodgkin lymphoma	1
